# FAMILY FLYNN EFFECTS AND LINKS TO MIDDLE-AGE HEALTH OUTCOMES

**DOI:** 10.1093/geroni/igac059.2256

**Published:** 2022-12-20

**Authors:** Linda Wänström, Patrick O'Keefe, Sean Clousten, Frank D Mann, Graciela Muniz Terrera, Stacey Voll, Scott Hofer, Joeseph Rodgers

**Affiliations:** Linkoping University, Linkoping, Ostergotlands Lan, Sweden; Oregon Health and Science University, Portland, Oregon, United States; Stony Brook University, Stony Brook, New York, United States; Stony Brook University, New York, New York, United States; Ohio University, Athens, Ohio, United States; University of Victoria, Victoria, British Columbia, Canada; University of Victoria, Victoria, British Columbia, Canada; Vanderbilt University, Nashville, Tennessee, United States

## Abstract

The Flynn effect (Flynn, 1984; 1987) refers to increases in cognitive performance, for later-born cohorts. It has been documented globally, occurring for more than a century. In a meta-analysis, Pietschnig and Voracek (2015) noted that the effect may be even stronger in adults than in children, though little research has addressed this topic (or its implications) for aging adults. Similarly, overall life-time health has improved, and incidences of cognitive impairment have decreased during the last two decades (Clouston et al., 2021). Using multilevel growth curve models, we found family Flynn effects in the National Longitudinal Survey of Youth; children in families with later-born mothers, and later-born first children, had higher PIAT math scores, and steeper developmental slopes. Although the link from childhood and adolescent cognitive function to later life outcomes has been well studied, research that takes advantage of the Flynn effect to facilitate interpreting that link is lacking. Clouston et al. (2021) emphasized the value of the Flynn effect in investigating links between childhood cognitive functioning and later adult Alzheimer’s disease and related dementia (ADRD) risks. We linked our family level results to middle-age maternal health outcomes (factors that are related to ADRD risks). Canonical correlation analyses showed that mothers (at ages 40+ and 50+) from families with higher score levels and slopes tended to have better mental and physical health. Our results, showing a Flynn effect in child and adolescence scores, at the family level, with links to adult health, persisted after controlling for a known selection bias.

